# Network Models to Enhance Automated Cryptocurrency Portfolio Management

**DOI:** 10.3389/frai.2020.00022

**Published:** 2020-04-24

**Authors:** Paolo Giudici, Paolo Pagnottoni, Gloria Polinesi

**Affiliations:** ^1^Department of Economics and Management, University of Pavia, Pavia, Italy; ^2^Department of Economics and Social Sciences, Universitá Politecnica delle Marche, Ancona, Italy

**Keywords:** cryptocurrencies, correlation networks, network centrality, portfolio optimization, random matrix theory, minimal spanning tree

## Abstract

The usage of cryptocurrencies, together with that of financial automated consultancy, is widely spreading in the last few years. However, automated consultancy services are not yet exploiting the potentiality of this nascent market, which represents a class of innovative financial products that can be proposed by robo-advisors. For this reason, we propose a novel approach to build efficient portfolio allocation strategies involving volatile financial instruments, such as cryptocurrencies. In other words, we develop an extension of the traditional Markowitz model which combines Random Matrix Theory and network measures, in order to achieve portfolio weights enhancing portfolios' risk-return profiles. The results show that overall our model overperforms several competing alternatives, maintaining a relatively low level of risk.

## 1. Introduction

FinTech innovations are rapidly expanding nowadays, with applications including payments, lending, insurance and asset management, among others. Two technical reports from the Financial Stability Board (FSB) (FSB, 2017a,b)—establish several key drivers for FinTech, i.e., the shift of consumer preferences on the demand side, the change of financial regulations on the supply side and the technology evolution.

In this context, services of automated financial consulting are widely spreading and, in particular robo-advisors^1^. They are supposed to match the investors' risk profile with specific class of financial assets and thereby build an efficient portfolio allocation for each specific client. However, the mechanisms underlying the portfolio construction are often obscure, as well as they arguably do not properly take into account for multivariate dependencies across securities which are key to achieve diversification and, therefore, mitigate financial risk. This is particularly true when dealing with peculiarly volatile markets, such as the cryptocurrency one, which could be one of the future target market of robo-advisors, given its rapidly growing influence in the financial world.

Indeed, after its introduction by Nakamoto (2008), Bitcoin was launched online in 2009 and paved the way for many other cryptocurrencies. As a matter of fact, as of 17 October 2019, the cryptocurrency market capitalization amounts to ~220 billion USD, with a daily trading volume of roughly 52 billion USD.

Along with descriptive and qualitative studies, many researches dealt with quantitative analysis applied to the cryptocurrency market. In particular, a stream of research focuses on price discovery on Bitcoin markets, aiming to determine which are the leaders and followers of the Bitcoin^1^ price formation process (see Brandvold et al., 2015; Pagnottoni and Dimpfl, 2018; Giudici and Abu-Hashish, 2019). Other related researches studied the interconnectedness and spillover in the cryptocurrency market (such as Corbet et al., 2018b; Giudici and Pagnottoni, 2019a,b). Another important area regards the study of Bitcoin derivatives—i.e., options and futures written on Bitcoin, with studies conducted by Corbet et al. (2018a), Baur and Dimpfl (2019), Giudici and Polinesi (2019), and Pagnottoni (2019).

From a methodological viewpoint, we base our analysis on an important stream of literature, which focuses on stock and financial networks built on correlation matrices. The seminal paper by Mantegna (1999) uses correlation matrices to infer the hierarchical structure of stock markets, deriving a distance measure based on correlation matrices and building the so called Minimal Spanning Tree (MST), a graphical representation able to connect assets which are similar in terms of returns in a pairwise manner. After that, a research by Tola et al. (2008) uses the Random Matrix Theory (RMT) together with several clustering techniques and show that this significantly lowers portfolio risks. Subsequently, other papers about portfolio construction involving the network structure of financial assets followed (see Zhan et al., 2015; León et al., 2017; Raffinot, 2017; Ren et al., 2017).

To the best of our knowledge, there are no papers yet that exploit network topologies to build portfolios composed by cryptocurrencies. We fill this gap proposing a model that exploits the network structure of cryptocurrencies to provide a portfolio asset allocation that well compares with traditional ones. Following Mantegna (1999) we use Markowitz' asset allocation as a benchmark, and we check whether our proposal is able to improve on it, in terms of risk/return profile.

Indeed, the originality of the current paper is 2-fold. From a methodological point of view, we improve the traditional (Markowitz, 1952) portfolio allocation strategy by means of RMT and MST and by taking network centralities specifically into account. Moreover, throughout this technique we are able to set a parameter of systemic risk aversion that investors can tune to better match their investment strategies with their own risk profile. From an empirical viewpoint, we apply our methodology to data coming from a nascent and highly volatile market, i.e., the cryptocurrency one. This is particularly interesting, as the cryptocurrency market is rapidly expanding and its opportunities due to the high uncertainty (and volatility) around it are quite appealing, and thus a greater number of investors will likely enter it in the short run.

Our empirical findings confirm the effectiveness of our model in achieving better cumulative portfolio performances, while keeping a relatively low level of risk. In particular, we show that our proposed model which employs RMT, MST and centrality measures rapidly adapts to market conditions, and is able to yield satisfactory performances during bull market periods. During bear market periods—instead—our Network Markowitz model employing RMT and MST realizes the best performances, protecting investors from relatively high losses which are instead generated by many other asset allocation strategies tested. Furthermore, the riskiness of our strategy is still lower than most of the competing model we analyze. These outcomes suggest that a sound combination of the proposed models should be employed in order to achieve an efficient cryptocurrency allocation strategy, which could be also used as robo-advisory toolboxes to improve automated financial consultancy.

The paper proceeds as follows. Section 2 presents our methodology and, particularly, the Random Matrix Theory, the Minimal Spanning Tree and the portfolio construction. Section 3 illustrates our empirical results. Section 4 concludes.

## 2. Methodology

### 2.1. Random Matrix Theory

Random Matrix Theory (RMT) is widely employed in several fields, such as quantum mechanics (Beenakker, 1997), condensed matter physics (Guhr et al., 1998), wireless communications (Tulino et al., 2004), as well as economics and finance (Potters et al., 2005). This technique is able to remove the noise component from the pure signal which is embedded into correlation matrices.

The algorithm tests subsequent empirical eigenvalues of the correlation matrix: λ_*k*_ < λ_*k*+1_; *k* = 1, …, *n*, against the null hypothesis that they are equal to the eigenvalues of a random Wishart matrix $\text{R}=\frac{1}{T}\text{A}{\text{A}}^{T}$ of the same size, being **A** a *N* × *T* matrix containing *N* time series of length *T*. The elements of **A** are *i.i.d*. random variables, with zero mean and unit variance.

Marchenko and Pastur (1967) show that as *N* → ∞ and *T* → ∞, and the ratio $Q=\frac{T}{N}\geq 1$ is fixed, there is convergence of the sample eigenvalues' density to:

(1) $$\begin{array}{l} f\left(\lambda \right)=\frac{T}{2\pi }\frac{\sqrt{\left(\lambda_{+}-\lambda \right)\left(\lambda -\lambda_{-}\right)}}{\lambda }, \end{array}$$

with λ ∈ (λ_−_, λ_+_), $\lambda_{\pm }=1+\frac{1}{Q}\pm 2\sqrt{\frac{1}{Q}}$.

Provided that, if λ_*k*_ > λ_+_ the null hypothesis is rejected from the *k*-th eigenvalue onwards. Hence, through a singular value decomposition the RM approach builds up a filtered correlation matrix (see Eom et al., 2009).

In our specific case, consider the continuous log return time series *r*_*i*_ of a generic cryptocurrency *i* at any time point *t*. i.e.,

(2) $$\begin{array}{l} r_{i}^{t}=logP_{i}^{t}-logP_{i}^{t-1}, \end{array}$$

where $P_{i}^{t}$ is the price of the cryptocurrency *i* at time *t*.

Considering a bunch of *N* cryptocurrency return time series, let **C** be the *N* × *N* correlation matrix of the cryptocurrency return time series. The random matrix approach filters the correlation matrix, thus obtaining a new matrix C^*^ as:

(3) $$\begin{array}{l} C^{*}\;=V\text{Λ}V^{T}, \end{array}$$

with

$$\begin{array}{l} \Lambda =\{\begin{array}{ll} 0 & \lambda_{i}<\lambda_{+}\\ \lambda_{i} & \lambda_{i}\geq \lambda_{+} \end{array} \end{array}$$

and **V** being the matrix of the deviating eigenvectors linked to the eigenvalues which are larger than λ_+_.

### 2.2. The Minimal Spanning Tree

In order to simplify the relationships given by the filtered correlation matrix C^*^ obtained from the random matrix approach, we apply the Minimal Spanning Tree representation of the cryptocurrency return time series. This is consistent with the literature on stock similarities (i.e., Mantegna and Stanley, 1999; Bonanno et al., 2003; Spelta and Araújo, 2012).

Given the filtered correlation matrix obtained in the step above, we may derive an Euclidean distance for each pairwise correlation element in the matrix, i.e.,

(4) $$\begin{array}{l} d_{ij}=\sqrt{2-2c_{ij}^{*}}, \end{array}$$

where $c_{ij}^{*}$ is a generic element (*i, j*) of the matrix C^*^, with *i, j* = 1, …, *N*. Each pairwise distance can be inserted in the so-called distance matrix **D** = {*d*_*ij*_}. The MST algorithm is able to reduce the number of links between the assets from $\frac{N\left(N-1\right)}{2}$ to *N* − 1 linking each node to its closest neighbor. In particular, we initially consider *N* clusters associated to the *N* cryptocurrencies and, at each subsequent step, we merge two generic clusters *l*_*i*_ and *l*_*j*_ if:

$$\begin{array}{l} d\left(l_{i},l_{j}\right)=\min\left\{d\left(l_{i},l_{j}\right)\right\}, \end{array}$$

with the distance between clusters being defined as:

$$\begin{array}{l} \hat{d}\left(l_{i},l_{j}\right)=\min\left\{d_{pq}\right\}, \end{array}$$

being *p* ∈ *l*_*i*_ and *q* ∈ *l*_*j*_. This procedure is iteratively repeated until we remain with just one cluster at hand.

Moreover, with the aim of explaining the evolution of relationships evolve over time, Spelta and Araújo (2012) proposed the so-called residuality coefficient, which compares the relative strength of the connections above and below a threshold distance value, i.e.,

(5) $$\begin{array}{l} R=\frac{\underset{d_{i,j}>L}{\sum }d_{i,j}^{-1}}{\underset{d_{i,j}\leq L}{\sum }d_{i,j}^{-1}} \end{array}$$

with *L* being the highest threshold distance value ensuring connectivity of the MST. Intuitively, the residuality coefficient *R* increases when the number of links increases—meaning the network becomes more sparse, and viceversa lowers with decreasing number of links.

### 2.3. Network Centrality Measures

In this paper we employ of centrality measures in order to develop a portfolio allocation that takes into account the centrality of a node (cryptocurrency) in the system. Network theory includes several centrality measures, such as the degree centrality, counting how many neighbors a node has, as well centrality measures based on the spectral properties of graphs (see Perra and Fortunato, 2008). Among the spectral centrality measures we remark Katz's centrality (see Katz, 1953), PageRank (Brin and Page, 1998), hub and authority centralities (Kleinberg, 1999), and the eigenvector centrality (Bonacich, 2007).

In this paper we employ of the eigenvector centrality, as it measures the importance of a node in a network by assigning relative scores to all nodes in the network. Relative scores are based on the principle that being connected to few high scoring nodes contributes more to the score of the node in question than equal connections to low scoring nodes. In other words, considering a generic node *i*, the centrality score is proportional to the sum of the scores of all nodes which are connected to it, i.e.,

(6) $$\begin{array}{l} x_{i}=\frac{1}{\lambda }\overset{N}{\underset{j=1}{\sum }}\hat{d_{i,j}}x_{j} \end{array}$$

where *x*_*j*_ is the score of a node *j*, $\hat{d_{i,j}}$ is the element (*i, j*) of the adjacency matrix of the network, λ is a constant. The equation from above can be rewritten in a compact form as:

(7) $$\begin{array}{l} \hat{\text{D}x}=\lambda \text{x} \end{array}$$

where $\hat{\text{D}}$ is the adjacency matrix, λ is the eigenvalue of the matrix $\hat{\text{D}}$, with associated eigenvector **x**, a vector of scores of dimension *N*, meaning one element for each node. Note that as our networks are based on distances between returns, the higher the centrality measure associated to a node, the more the node behaves dissimilarly with respect to the other nodes in the network.

### 2.4. Portfolio Construction

Asset correlations are key items in investment theory and risk measurement, in particular for optimization problems as in the case of the widely known portfolio theory described by Markowitz (1952). As a consequence, correlation based graphs are useful tool to build optimal investment strategies. In this subsection we show how portfolio construction can be enhanced by means of a combination of the RMT, MST, and network centrality measures described above.

Several researches have investigated the relationship between the network structure of financial assets and portfolio strategies. The study (Onnela et al., 2003) shows how a portfolio constructed via Markowitz theory is mainly composed by assets that lie in the periphery of the asset network structure, i.e., outer node assets, and not in its core. Pozzi et al. (2013) find that peripheral assets in the network yield to better performances and lower portfolio risk with respect to central ones. Peralta and Zareei (2016) show that the centrality of assets within a network are negatively related with the optimal weights obtained through the Markowitz technique. Building on that, Vỳrost et al. (2018) conclude that asset allocation strategies including the network structure of financial asset are able to improve a portfolio's risk-return profile.

Another stream of literature focused on proposing alternative portfolio allocation strategies based on the network structure of financial assets. To illustrate, Plerou et al. (2002) and Conlon et al. (2007) use the random matrix theory to filter the correlation matrix to be inserted in the Markowitz minimization problem, while Tola et al. (2008) add the MST obtaining improvements with respect to the raw model.

In the present context we aim to study the differences in the risk-return profiles of our strategy, which includes topological measures in the optimization problem, with respect to the traditional Markowitz model, possibly yielding to better risk-return characteristics of the portfolios. The originality of our approach builds on the fact that we do not only use RMT and MST as alternative approaches to quantify risk diversification, but we employ an extension of the traditional Markowitz method by including these techniques in the minimization problem. Indeed, in the present case we want to solve the following problem:

(8) $$\begin{array}{l} \underset{\text{w}}{\min}{\text{w}}^{\text{T}}\Sigma^{*}\text{w}+\gamma \overset{n}{\underset{i=1}{\sum }}x_{i}w_{i} \end{array}$$

subject to

$$\begin{array}{l} \{\begin{array}{l} \begin{array}{c} \sum_{i=1}^{n}w_{i}=1\\ \mu_{P}\geq \frac{\sum_{i=1}^{n}\mu_{i}}{n}\\ w_{i}\geq 0 \end{array} \end{array} \end{array}$$

where **w** is the vector of portfolio weights, being *w*_*i*_ the weight associated to the cryptocurrency *i*, Σ^*^ is the filtered variance-covariance matrix with generic element (*i, j*) represented by $\sigma_{i}\sigma_{j}c_{i,j}^{*}$, γ is the parameter representing the risk aversion of the investor, *x*_*i*_ is the eigenvector centrality associated with the cryptocurrency *i*, μ_*P*_ indicates the return of the portfolio and μ_*i*_ the return of the generic cryptocurrency *i*.

Generally speaking, portfolios built upon the traditional Markowitz theory are such that the risk is minimized for a given expected return, using as input the raw variance-covariance matrix of returns. In our case, the methodological improvement is 2-fold. Firstly, we modify the input variance-covariance matrix, which is filtered by both RMT and MST. Secondly, we add a component derived from the MST structure which relates to an extra risk component the investor may want to control for. Indeed, by modulating γ the investor can set its own level of risk aversion toward systemic risk specifically, and not just to the portfolio risk as in the Markowitz framework. As a matter of fact, being centralities inversely related with distances, a small value of γ yields to portfolios composed by less systemically risky cryptocurrencies, which generally lie in the peripheral part of the network. Conversely, a large value of γ makes the algorithm select more systemically relevant cryptocurrencies, meaning those who are in the center of the network structure. For the sake of completeness, we will test different values of the systemic risk aversion parameter in the course of the current application.

Starting from the cryptocurrency return time series, the steps of the algorithm can be summarized as follows:

1. Estimation of the filtered correlation matrix C^*^ by RMT
2. Reduction of the number of links in the filtered correlation matrix C^*^ by MST
3. Computation of the filtered variance-covariance matrix Σ^*^ associated to the filtered correlation matrix C^*^ in step 2
4. Computation of the eigenvector centralities *x*_*i*_
5. Computation of the portfolio weights by solving the minimization problem:

$$\begin{array}{l} \underset{\text{w}}{\min}{\text{w}}^{T}\Sigma^{*}\text{w}+\gamma \overset{n}{\underset{i=1}{\sum }}x_{i}w_{i}s.t.\{\begin{array}{l} \begin{array}{c} \sum_{i=1}^{n}w_{i}=1\\ \mu_{P}\geq \frac{\sum_{i=1}^{n}\mu_{i}}{n}\\ w_{i}\geq 0 \end{array} \end{array}\} \end{array}$$

The weights calculation finally yields to the portfolio returns which we use to evaluate the performance of our allocation method.

## 3. Empirical Findings

### 3.1. Data Description and Network Topology Analysis

In our empirical application we consider 10 time series of returns referred to cryptocurrencies traded over the period 14 September 2017–17 October 2019 (764 daily observations). In particular, we consider the first 10 cryptocurrencies in terms of market capitalization as of 17 October 2019^2^. To be precise, we analyze the return time series of the following cryptocurrencies: Bitcoin (BTC), Ethereum (ETH), Ripple (XRP), Tether (USDT), Bitcoin Cash (BCH), Litecoin (LTC), Binance Coin (BNB), Eos (EOS), Stellar (XLM), Tron (TRX).

We provide some basic descriptive statistics of our data in Table 1. From Table 1 one may notice that average daily returns are all close to zero, in line with the general economic theory regarding asset returns. However, the 10 cryptocurrencies exhibit different standard deviations, meaning that the variability in returns differs quite strongly among cryptocurrencies. To illustrate, USDT is the one showing the lowest relative variability; this is in line with the fact that this cryptocurrency is classified as stable coin, therefore its price should not deviate too much on a daily basis. On the other hand, TRX is the one showing the highest standard deviation; indeed, this particular cryptocurrency witnessed a period of high fluctuations during the considered sample period. As far as kurtosis is concerned, most of the cryptocurrencies exhibit values which reflects the non-Gaussian and heavy tailed behavior of their associated distribution. This is particularly true for XLM and XRP, whose kurtosis are relatively larger than the ones of the other time series.

**Table 1:** Summary statistics.

	**Mean**	**Std**	**Kurtosis**	**Skewness**
BTC	0.0009	0.04	3.35	−0.07
ETH	−0.0007	0.05	2.90	−0.33
XRP	0.0004	0.07	15.73	1.80
USDT	0.0000	0.01	4.28	0.22
BCH	−0.0011	0.08	6.47	0.49
LTC	−0.0003	0.06	8.02	0.66
BNB	0.0033	0.07	7.74	0.78
EOS	0.0017	0.07	3.93	0.60
XLM	0.0021	0.10	26.19	2.03
TRX	0.0021	0.15	13.15	0.66

*The table shows relevant summary statistics for the 10 cryptocurrencies considered related to the whole sample period, i.e., 13 September 2017–10 October 2019*.

**Figure 1:**
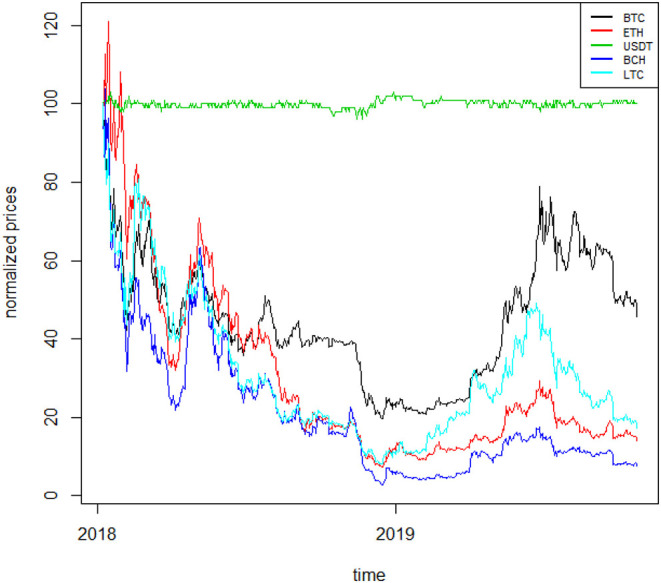
Normalized cryptocurrency price series I. This figure shows the normalized price series for five cryptocurrencies: XRP, BNB, EOS, XLM, TRX, relative to the period 7 January 2018–17 October 2019.

**Figure 2:**
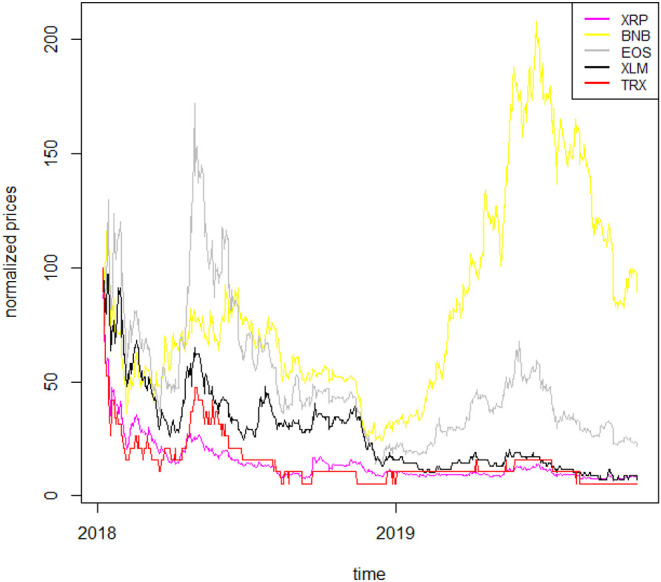
Normalized cryptocurrency price series II. The figure shows the normalized price series for five cryptocurrencies: BTC, ETH,USDT, BCH, LTC, relative to the period 7 January 2018–17 October 2019.

To better understand the dynamics of the cryptocurrency time series, we plot the normalized price series in Figures 1, 2,^3^. The two figures confirm well-known features of cryptocurrencies, such as their overall high volatility (with TRX being the most volatile), the stability of the stable coin (USDT) as well as the low liquidity that some of them exhibit (such as TRX).

In order to apply the filter through RMT, we divide the dataset into consecutive overlapping windows having a width *T* = 120 (4 trading months). We set the window step length to 1 week (7 trading days), which makes up a total of 93 weekly 4-months windows.

For each time window considered, we use 15 weeks of daily observations to estimate the model, while the last week is used for validation purposes. In other words, we compute 93 correlation matrices between the 10 cryptocurrency return time series, each one based on 15 weeks of daily returns and then filter them by means of the Random Matrix approach. Applying the Random Matrix filtering, correlation matrices are rebuilt considering only the eigenvectors corresponding to the deviating eigenvalues.

**Figure 3:**
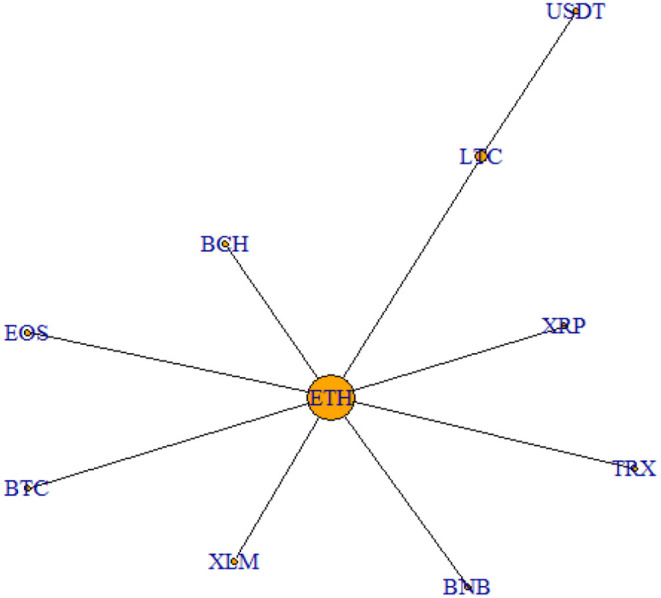
MST September 2017–January 2018. This figure shows the MST representation relative to the period of the speculative bubble.

**Figure 4:**
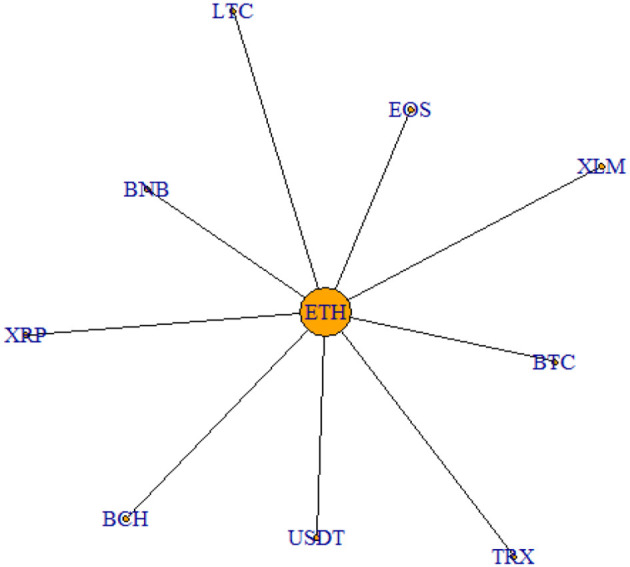
MST June 2019–October 2019. This figure shows the MST relative to the period June 2019–October 2019.

In order to have a better understanding of the links existing between cryptocurrencies, the filtered correlation matrices are then used to derive the MST representation over two main periods of interest. In particular, we plot the MST structure emerging from the period of the cryptocurrency price hype (September 2017–January 2018) in Figure 3, while the MST structure related to the latest trading period analyzed (June 2019–October 2019) in Figure 4.

As it is clear from the graph, the two networks show quite similar features. Indeed, ETH is the cryptocurrency which always lies in the center of the structure, indicating its central role in the cryptocurrency market. The only difference between the two graphical representations concerns USDT, which during the price hype is not connected directly to ETH as the other cryptocurrencies, but to LTC. This is linked to the fact that USDT is a stable coin and, therefore, behaves dissimilarly from the other cryptocurrencies considered, being it much less volatile. However, this difference in behavior levels out during the latest period, as it emerges from Figure 4.

**Figure 5:**
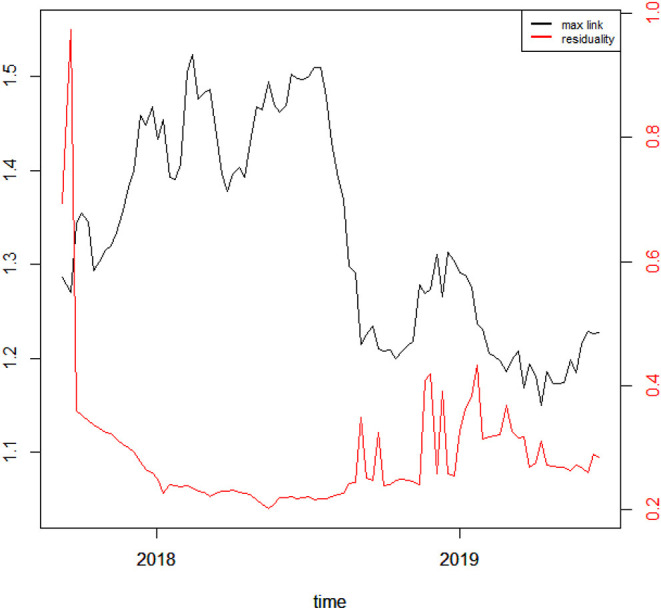
MST thresholds and residuality coefficients. The black line shows the Max link distance, while the red line shows the residuality coefficient, whose values are reported, respectively on the left and right y axis.

To better understand the dynamics of the MST among cryptocurrencies, we investigate the evolution of the links over time. Indeed, we compute two different measures: the Max link, i.e., the value of the maximum distance between two pairs of nodes in the tree, and the residuality coefficient, meaning the ratio between the number of links which are dropped and the number of those who are kept by the MST algorithm. The two metrics, computed over the whole sample period, are illustrated in Figure 5.

From Figure 5 one may notice that the Max link increases during the Bitcoin price hype and fluctuates around relatively large values until roughly mid 2018, meaning that during this period correlations between cryptocurrency returns are strongly misaligned. After that, the index bounces back toward its previous values and even below, suggesting that cryptocurrency returns start to behave more similarly during the latest period. Furthermore, the residuality coefficient increases during the very beginning of the sample period, while it sharply declines during the price hype phase. After the decrease, the coefficient stays quite stable and then gently increases not without fluctuations from mid 2018 to the end of the sample period. This suggests that the number of links until mid 2018 was quite limited, and therefore, returns misaligned, whereas the same number started to increase after that phase, meaning there were more connections and thus more synchronicity across cryptocurrency returns.

### 3.2. Portfolio Construction

In this subsection we illustrate the results related to the proposed portfolio strategies. The optimal portfolio weights are obtained through the constrained minimization of the objective function in Equation 8. For the sake of completeness, we use different values of the systemic risk aversion parameter γ, meaning γ = 0.005, 0.025, 0.05, 0.15, 0.7, 1. These values have been chosen, without loss of generality, to be representative of different aversion profiles. While γ = 0 indicates no aversion, γ = 1 indicates a high aversion, with systemic risk being given the same importance as non-systemic one.

We use fifteen weeks, i.e., to compute the optimal portfolio weights as described in section 2. We then use the last week associated to each window to evaluate the out-of-sample performance of our technique, meaning to compute the portfolio returns and, therefore, the resulting Profit & Losses. We then compute portfolio returns for the period 7 January 2018–17 October 2019, accounting for rebalancing costs, which are supposed to amount to 10 basis points.

**Figure 6:**
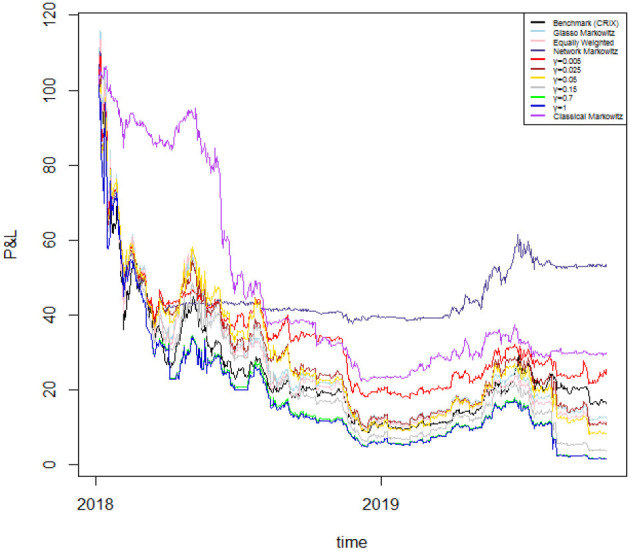
Performances of different portfolio strategies. The plot reports the profits and losses of a portfolio with initial value of 100 USD obtained by the CRIX benchmark index [Benchmark (CRIX)], the optimization using the Markowitz approach with the variance-covariance matrix filtered by Glasso (Glasso Markowitz), the naive portfolio (Equally Weighted), our optimization using RMT and MST applied to the variance-covariance matrix (Network Markowitz), our model based on different values of γ (γ=0.005, 0.025, 0.05, 0.15, 0.7, 1), and the standard Markowitz portfolio (Classical Markowitz). The portfolio values are plotted for the period 7 January 2018–17 October 2019.

**Figure 7:**
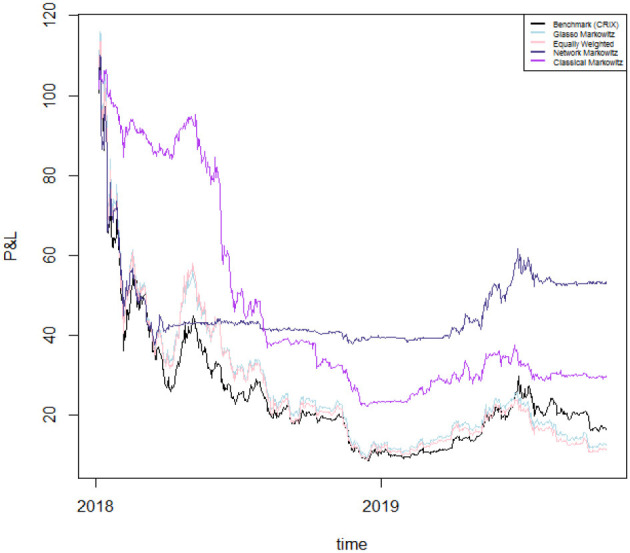
Performances of selected portfolio strategies. The plot reports the profit and losses of a portfolio with initial value of 100 USD obtained by the CRIX benchmark index [Benchmark (CRIX)], the optimization using the Markowitz approach with the variance-covariance matrix filtered by Glasso (Glasso Markowitz), the naive portfolio (Equally Weighted), our optimization using RMT and MST applied to the variance-covariance matrix (Network Markowitz) and the standard Markowitz portfolio (Classical Markowitz). The portfolio values are plotted for the period 7 January 2018–17 October 2019.

In Figure 6 we plot the returns of our investment strategies for the different values of γ mentioned above as well as for γ = 0 (Network Markowitz), meaning the results of the Markowitz portfolio strategy using the variance-covariance matrix filtered by RMT and MST. In doing so, we plot portfolio performances under the hypothesis of investing 100 USD at the beginning of the period, and examining how much is lost along time. The results of our strategies are compared with the performance of several strategies and indicators: the benchmark portfolio (CRIX^4^), the Markowitz portfolio with variance-covariance matrix filtered by the Glasso^5^ technique (Glasso Markowitz), the naive portfolio (Equally Weighted) and the traditional Markowitz portfolio (Classical Markowitz). To better highlight the results of our best proposed model, we plot the results only for a selection of portfolio strategies in Figure 7. To complement this information, we report the 4-months cumulative Profits and Losses of each of the considered strategy in Table 2.

**Table 2:** Cumulative Profits and Losses.

**Period**	**CRIX**	**GM**	**EW**	**CM**	**NW**	******γ = 0.005******	******γ = 0.025******	******γ = 0.05******	******γ = 0.15******	****γ = 0.7****	******γ = 1******
Jan-2018	−0.14	−0.13	−0.16	0.04	−0.22	−0.21	−0.26	−0.27	−0.36	−0.43	−0.43
May-2018	−0.67	−0.62	−0.60	−0.12	−0.79	−0.78	−0.73	−0.66	−0.83	−1.08	−1.10
Sep-2018	−1.37	−1.37	−1.43	−0.88	−0.83	−1.02	−1.24	−1.23	−1.40	−1.60	−1.64
Jan-2019	−1.85	−1.78	−1.78	−1.32	−0.87	−1.50	−1.86	−1.98	−2.19	−2.29	−2.31
May-2019	−1.35	−1.25	−1.27	−1.01	−0.74	−1.22	−1.33	−1.29	−1.44	−1.55	−1.57
Sep-2019	−0.99	−1.45	−1.49	−1.02	−0.54	−1.19	−1.34	−1.44	−1.86	−2.13	−2.15

*This table shows the cumulative 4-months Profits and Losses of portfolios under different strategies. Particularly, Profits & Losses are computed for the CRIX benchmark index (CRIX), the Glasso Markowitz (GM), the naive portfolio (EW), the Network Markowitz (NW), the classical Markowitz (CM), and the proposed models with different values of γ (γ = 0.005, 0.025, 0.05, 0.15, 0.7, 1). All values are expressed in percentage terms*.

Overall, we are considering a period in which the cryptocurrency market witnesses a down period—except for the first part of our analyzed timespan and several short periods consequently occurring. Therefore, as the market is not profitable during the studied period, we aim to achieve through our allocation strategies losses which are lower than those yielded by other competing methodologies.

On the one hand, during a first phase which lasts roughly until mid 2018, the traditional Markowitz portfolio seems to overperform the other portfolio allocation strategies. Indeed, the allocation by Markowitz' technique yields to positive (cumulative) returns until January 2018 and just slightly negative ones until May 2018, however still lower than the losses provided by the other strategies in absolute terms.

On the other hand, from September 2018 onwards all portfolios start providing strong negative returns. Indeed, the returns yielded by the portfolio constructed via Markowitz start to decline dramatically, together with those of the model including the systemic risk aversion parameter. This is because the latter model takes into account the centrality of the cryptocurrencies in the network and is therefore more adaptive to market conditions, regardless of whether they are favorable or not. Indeed it can be noticed that—overall—during bull market periods our model taking into account for risk aversion reacts very fast to upward movements and yields to good cumulative performances; conversely, during down market periods, the same model yields to worse relative performances due to declining market conditions.

However, during the second half of our sample period our proposed model with the systemic risk aversion parameter γ set to 0 (Network Markowitz) clearly overwhelms the other portfolio allocation strategies. To illustrate, if we look at the cumulative performance of the above mentioned method, we can see that it more than halves losses with respect to the equally weighted portfolio, to the Glasso Markowitz portfolio and to all portfolios including a risk aversion parameter γ > 0. Moreover, it almost halves the losses with respect to the benchmark index (CRIX) and to the traditional Markowitz methodology. This suggests that this model is capable to provide a stronger coverage for losses in case of down market periods with respect to all other considered asset allocation strategies^6^.

In Table 3 we compute the 4-months Value at Risk (VaR) with a confidence level of 0.05% for the benchmark index (CRIX), the equally weighted portfolio, our Network Markowitz portfolio, the Glasso Markowitz and the traditional Markowitz portfolios. This is done in order to compare, together with cumulative returns, the potential riskiness of our strategy with respect to the alternative portfolio allocation methods considered.

**Table 3:** VaR.

**Period**	**CRIX**	**EW**	**NW**	**GM**	**CM**
Jan-2018	0.11	0.13	0.15	0.14	0.03
May-2018	0.04	0.05	0.02	0.05	0.03
Sep-2018	0.11	0.11	0.10	0.12	0.02
Jan-2019	0.07	0.10	0.05	0.07	0.01
May-2019	0.04	0.02	0.03	0.02	0.04
Sep-2019	0.05	0.05	0.02	0.05	0.01

*This table shows the 4-months Value at Risk of portfolios under different strategies for a confidence interval of 95%. In particular, the VaR is computed for the CRIX benchmark index (CRIX), the naive portfolio (EW), the Network Markowitz (NW), the Glasso Markowitz (GM), and the classical Markowitz (CM). All values are expressed in absolute terms multiplied by a scale factor of 100*.

Table 3 shows that, except for the price hype period, our proposed Network Markowitz approach generally yields to lower values at risk with respect to the benchmark index (CRIX), the naive portfolio and the Glasso Markowitz. The aforementioned model is instead more risky than the traditional Markowitz model, although the latter, overall, yields too far way larger negative returns. In general, the riskiness of our strategy seems to be quite satisfactory with respect to the alternative allocation strategies analyzed.

To further support our conclusions, Table 4 presents the Sharpe ratio under the different strategies.

**Table 4:** Sharpe ratio.

**Period**	**GM**	**EW**	**CM**	**NW**	******γ = 0.005******	******γ = 0.025******	******γ = 0.05******	******γ = 0.15******	******γ = 0.7******	******γ = 1******
Jan-2018	−0.05	−0.05	−0.03	−0.13	−0.12	−0.08	−0.06	−0.08	−0.09	−0.10
May-2018	−0.14	−0.14	−0.19	−0.03	−0.04	−0.08	−0.09	−0.08	−0.07	−0.07
Sep-2018	−0.10	−0.09	−0.17	−0.04	−0.17	−0.17	−0.20	−0.20	−0.18	−0.17
Jan-2019	0.10	0.09	0.11	0.09	0.06	0.08	0.11	0.12	0.12	0.12
May-2019	−0.02	−0.02	0.01	0.08	0.02	0.02	−0.00	−0.03	−0.04	−0.04
Sep-2019	−0.06	−0.06	−0.03	0.03	0.07	−0.11	−0.14	−0.14	−0.14	−0.14

*This table shows the 4-months values of Sharpe ratio of portfolios under different strategies. In particular, the SR is computed for the Glasso Markowitz (GM), the naive portfolio (EW), the classical Markowitz (CM), the Network Markowitz (NW), and for all the value of γ*.

Table 4 gives further evidence to support our conclusions: the proposed Network Markowitz approach yields better Sharpe Ratios.

To strengthen the robustness of our conclusions, Table 5 presents the Rachev ratio, with a confidence level of 10%, under the different strategies. The Rachev ratio is a useful supplement of the Sharpe ratio, when data is non-symmetric, as in our context. It is calculated as the ratio between an extreme gain and an extreme loss.

**Table 5:** Rachev ratio.

**Period**	**GM**	**EW**	**CM**	**NW**	******γ = 0.005******	******γ = 0.025******	******γ = 0.05******	******γ = 0.15******	******γ = 0.7******	******γ = 1******
Jan-2018	0.74	0.75	0.63	0.64	0.69	0.77	0.79	0.78	0.77	0.99
May-2018	0.73	0.75	0.95	0.83	0.74	0.77	0.83	0.87	0.87	0.55
Sep-2018	0.81	0.84	0.87	0.61	0.80	0.75	0.76	0.80	0.80	0.48
Jan-2019	1.16	1.11	1.47	1.24	1.34	1.36	1.39	1.40	1.40	1.26
May-2019	0.80	0.80	1.05	0.97	0.93	0.84	0.75	0.72	0.72	0.98
Sep-2019	0.75	0.78	1	1.14	0.43	0.38	0.38	0.38	0.37	0.78

*This table shows the 4-months values of Rachev Ratio (RR) of portfolios under different strategies. In particular, the RR is computed for the Glasso Markowitz (GM), the naive portfolio (EW), the classical Markowitz (CM), the Network Markowitz (NW), and for all the value of γ*.

Table 5 shows that the Network Markowitz approach yields the best performances in the initial and final periods, and the Classic Markowitz in all other periods. The other strategies generally show worse performances. This is consistent with our previous findings, and with the fact that the Rachev ratio takes higher values during periods characterized by decreasing returns, such as the quarter preceding January 2019.

Overall, we cannot say that the proposed model overperforms traditional approach (such as Glasso Markowitz and Classical Markowitz). It does so in certain periods and according to certain risk aversion parameterizations.

**Figure 8:**
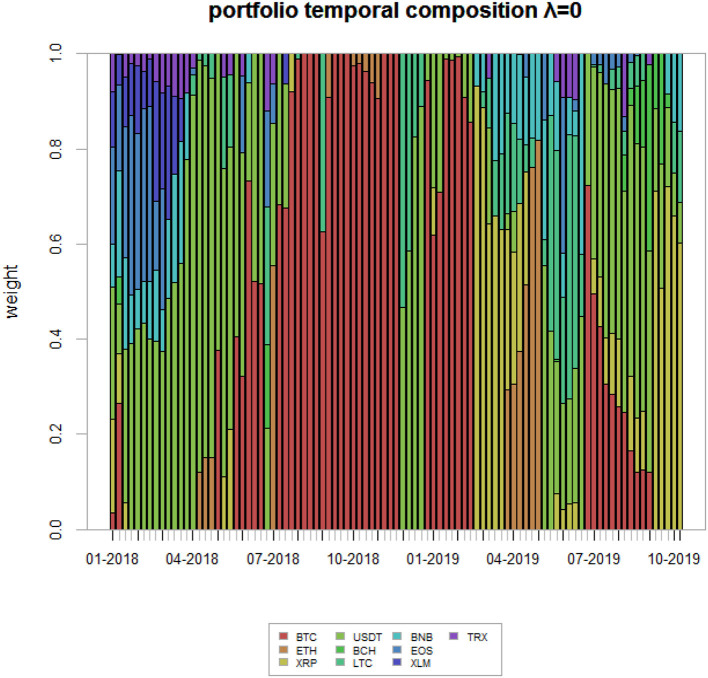
Winning strategy portfolio weights. The figure shows the portfolio weights associated to the winning strategy—i.e., the Network Markowitz (NW)—for the analyzed time period.

For the sake of completeness, we plot the portfolio weights of the winning strategy over the evaluation time horizon in Figure 8. As one can clearly see, the composition of the portfolio varies quite much over time. Indeed, during the first period of the sample, approximately until February 2018, the portfolio is composed by various assets, with USDT gaining a high share over time, being it the most stable across all. After that, BTC is the cryptocurrency which is mostly selected by our algorithm, roughly until October 2018 (with some exceptions), as it is considered a proxy of the whole market. Finally, the algorithm selects different cryptocurrency compositions until the end of the sample, being the latter a highly uncertain period for the market.

Last, we present, for comparison purposes, the portfolio weights associated with γ = 1.

**Figure 9:**
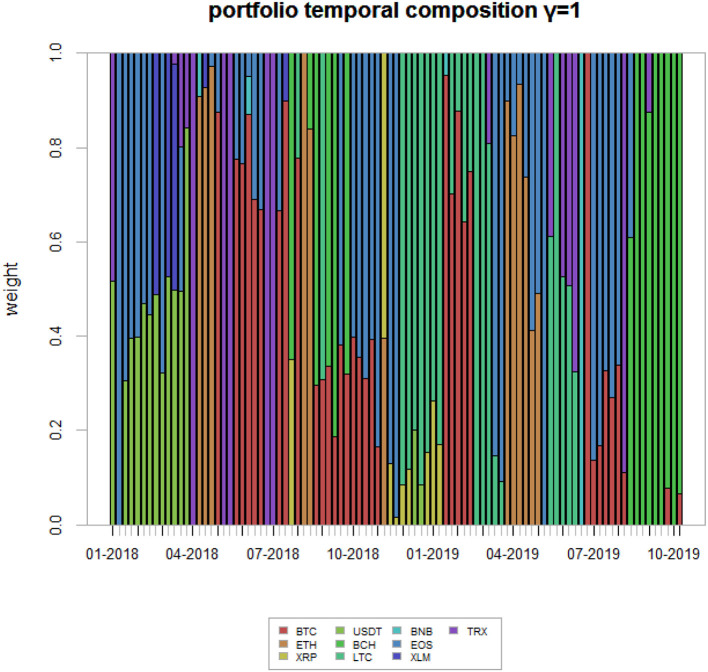
Highly risk adverse strategy portfolio weights. This figure shows the portfolio weights associated to a highly systemic risk adverse strategy—γ = 1—for the analyzed time period.

While Figure 8 gives the weights relative to the situation of no systemic risk aversion, Figure 9 gives the weight corresponding to a very high systemic risk aversion, in which it has the same importance as non-systemic risk.

## 4. Conclusions

In this paper we have proposed a methodology that aims to build an allocation strategy which is suitable for highly volatile markets, such as cryptocurrency ones. In particular, we have applied our models to a set of 10 cryptocurrency return time series, selected in terms of market capitalization. We have shown that the use of network models can enhance portfolios' risk-return profiles and mitigate losses during down market periods.

We have demonstrated how the use of centrality measures, together with tuning an investor's systemic risk aversion, is a suitable methodology to make profits during bull market periods, as this method is rapidly adaptive to market conditions. We have also shown that, to protect investors from losses during bear market periods, the combination of Random Matrix Theory and Minimal spanning trees can yield to acceptable risk-return profiles and/or mitigate losses.

Our empirical findings show that, overall, the proposed method is acceptable, even during downturn periods. However, we cannot claim that this proposed model should always be used in automated consultancy. It should always be compared with competing alternatives, according to different market conditions and different risk aversions.

We strongly believe that the proposed model should be further tested in different contexts. For this purpose, we provide at https://www.fintech-ho2020.eu a link to the used data and software, so the proposed methods can be fully reproduced. The software is written in the R language, and allows the methods to be extended to other data and contexts.

Further research should involve, besides the application to other contexts, the consideration of different base portfolio allocation models. We have used Markowitz' as is the most employed by robot advisory platforms.

## Data Availability Statement

The datasets analyzed for this study can be found in Coinmarketcap (https://coinmarketcap.com/). The software used in this article is not publicly available because based on a proprietary source. Requests to access the datasets should be directed to Gloria Polinesi (glopol@hotmail.it).

## Author Contributions

The paper was written in close collaboration between the authors. However, sections 2 and 4 have been written by GP, section 1 and 3 by PP, while PG has supervised and coordinated the work.

## Conflict of Interest

The authors declare that the research was conducted in the absence of any commercial or financial relationships that could be construed as a potential conflict of interest.

## Footnotes

^1^An article published on “Statista” in 2019 states that assets under management in the robo-advisory segment amounts to roughly 981 billion USD, as well as that they are expected to grow at an annual growth rate (CAGR 2019–2023) of 27% (source: https://www.statista.com/outlook/337/100/robo-advisors/worldwide).

^2^We exclude Bitcoin SV (BSV) in order to achieve a sufficiently large timespan, meaning a more than 2-years time period.

^3^We split the plot in two different figures for scale reasons.

^4^The CRIX is a cryptocurrency market index following the Laspeyres methodology for the construction of indexes. More information about CRIX can be found at https://thecrix.de/

^5^The sparsity parameter ρ has been set to 0.01, as in the reference paper by Friedman et al. (2008).

^6^A sensitivity analysis reported in the Appendix confirms that results are robust with respect to different choices of the starting points and rolling estimation windows.
