# Supplementary material for: Network Models to Enhance Automated Cryptocurrency Portfolio Management
Source: Front Artif Intell. 2020 Apr 24;3:22. doi: 10.3389/frai.2020.00022 (PMC7861261; doi:10.3389/frai.2020.00022)
Supplement: Supplementary file 1 [file Presentation_1.pdf]

Appendix

To further investigate the robustness of our results, we have performed a twofold sensitivity analysis. On the one hand, we have shifted the starting point of investing 4, 8, and 12 weeks into the future and computed cumulative portfolio returns again. In this analysis we have maintained our rolling 15 weeks window for correlation estimation. Outcomes are represented in Figure 1, panels a, b and c, respectively. On the other hand, we have performed the analysis again, but using a 19-week estimation window, meaning from rolling 4 months to rolling 5 months, changing both the starting point of investing and the window for correlation estimation. Results are shown in Figure 2. In both cases, outcomes suggest that our results are robust even with changing shifting starting points and different rolling estimation windows. Indeed, performances show that the Network Markowitz model outperforms the competitive strategies even with varying the starting point of the analysis and the estimation window choice. Moreover, the strategy results with profits rather than losses in some cases when shifting the investment starting point.

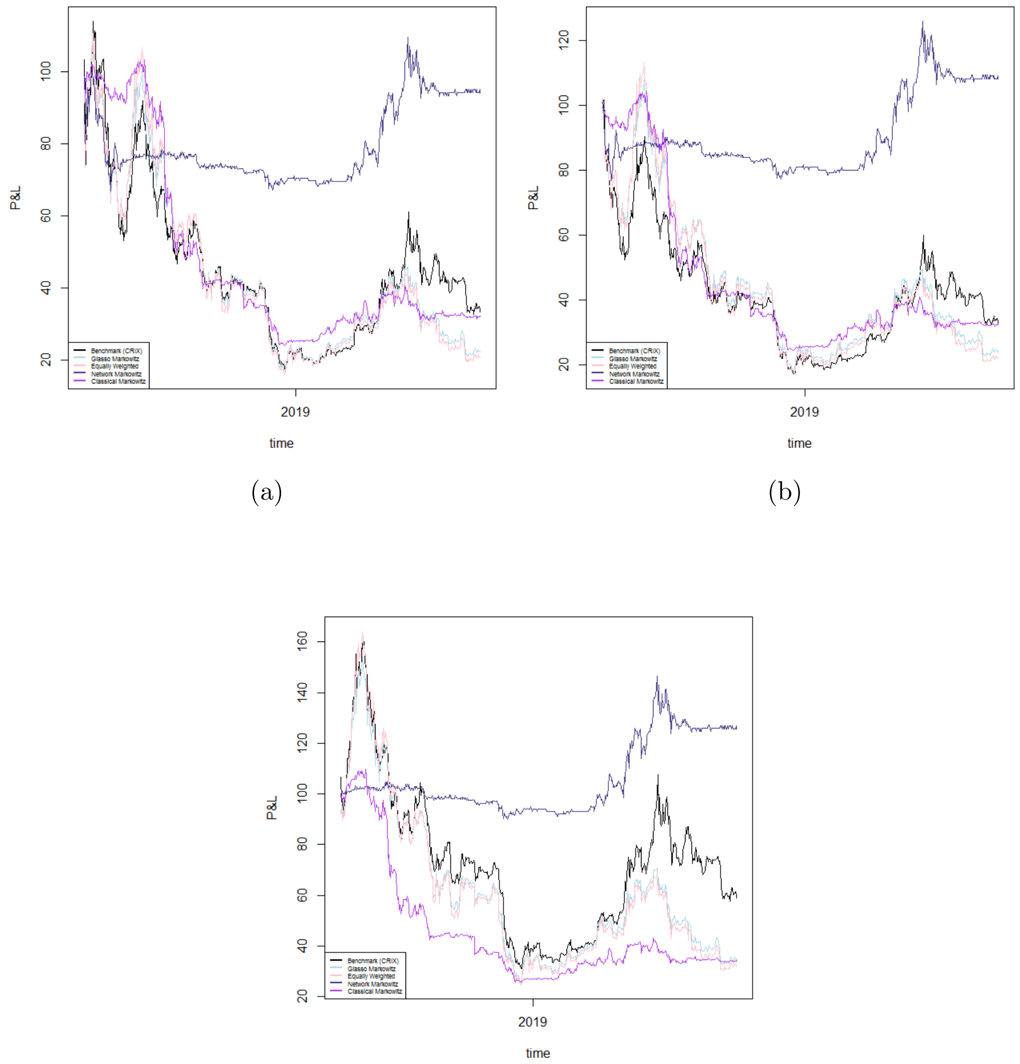

(a) (b)

(c)

Figure 1: **Cumulative returns for selected portfolio strategies with shifting starting points**. The plot illustrates the profit and losses of a portfolio with initial value of 100 USD obtained by the CRIX benchmark index (Benchmark (CRIX)), the optimization using the Markowitz approach with the variance-covariance matrix filtered by Glasso (Glasso Markowitz), the naive portfolio (Equally Weighted), our optimization using RMT and MST applied to the variance-covariance matrix (Network Markowitz) and the standard Markowitz portfolio (Classical Markowitz). Starting points are shifted by 4 weeks (panel a), 8 weeks (panel b) and 12 weeks (panel c) for sensitivity purposes.

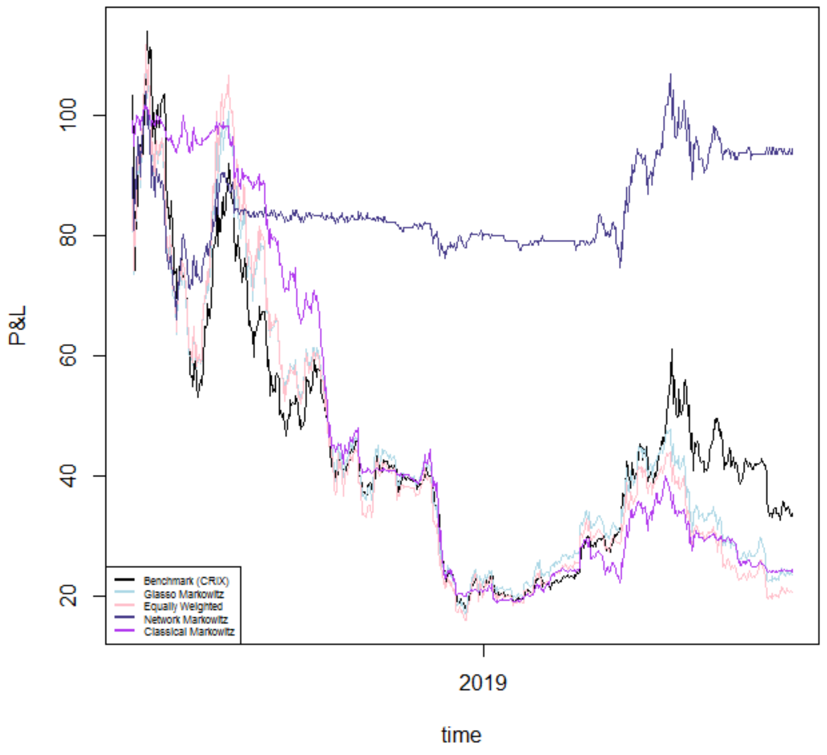

Figure 2: **Cumulative returns for selected portfolio strategies with different rolling estimation windows**. The plot reports the profit and losses of a portfolio with initial value of 100 USD obtained by the CRIX benchmark index (Benchmark (CRIX)), the optimization using the Markowitz approach with the variance-covariance matrix filtered by Glasso (Glasso Markowitz), the naive portfolio (Equally Weighted), our optimization using RMT and MST applied to the variance-covariance matrixt (Network Markowitz) and the standard Markowitz portfolio (Classical Markowitz). We use a rolling estimation window of 19 weeks for sensitivity purposes.
